# Ethical concerns and strategies for implementing artificial intelligence in healthcare: a review of empirical studies

**DOI:** 10.1186/s12910-026-01404-8

**Published:** 2026-02-07

**Authors:** Betelhem Zewdu Wubineh, Fitsum Gizachew Deriba, Fikadu Wayesa Gemeda

**Affiliations:** 1 Faculty of Information and Communication Technology, Wroclaw University of Science and Technology, Wroclaw, Poland; 2 School of Computing, University of Eastern Finland, Joensuu, Finland; 3 School of Computing and Informatics, Wachemo University, Hosaena, Ethiopia; 4 Space Science and Geospatial Institute, Addis Ababa, Ethiopia

**Keywords:** Artificial intelligence, Challenges in healthcare, Ethical concerns, Ethical strategies, Healthcare, Systematic review

## Abstract

Artificial intelligence (AI) is profoundly transforming the healthcare landscape, presenting unprecedented opportunities to enhance patient care and clinical outcomes. However, the rapid integration of AI technologies has raised significant ethical concerns, requiring rigorous scrutiny to ensure their responsible and equitable use. This study aimed to explore the ethical considerations and strategies related to the implementation of AI in healthcare through a systematic review. A systematic search identified 243 publications published between 2019 and 2025 that were initially identified. After applying inclusion and exclusion criteria, 22 papers were selected for final synthesis to assess ethical concerns and strategies related to AI in healthcare. The analysis identified key ethical concerns, categorizing them into six distinct groups: (1) Transparency and Trust, (2) Bias and Fairness, (3) Privacy and Data Security, (4) Accountability and Responsibility, (5) Ethical and Moral, (6) Regulatory and Legal. Additionally, several ethical strategies were identified in the implementation of AI systems, including adherence to ethical principles, standards, and frameworks; transparency and bias mitigation; monitoring and auditing of AI systems; and stakeholder involvement and governance in decision-making processes. This review emphasizes the importance of addressing these ethical concerns to ensure the successful implementation of AI technologies in healthcare. The findings provide valuable insights and recommendations for stakeholders, including developers, healthcare professionals, and policymakers, to guide the ethical deployment of AI decision support systems in healthcare.

**Supplementary Information:**

The online version contains supplementary material available at 10.1186/s12910-026-01404-8.

## Background

Artificial intelligence (AI) has advanced in recent years, influencing people’s lives in various sectors. In healthcare, AI techniques such as machine learning and deep learning are widely used in diagnosis, risk prediction, and clinical decision support [1, 2]. These AI applications serve numerous purposes in the health domain, including health risk analyses, health information management, virtual health assistance, and clinical decision-making [3]. Despite this potential, studies have identified persistent challenges in AI implementation, such as the lack of ethical principles, the limited healthcare-specific applicability of existing ethical frameworks, and insufficient examination of practical AI applications in clinical settings [4, 5].

To address these challenges, many countries and international bodies advocate for ethical standards and principles for implementing AI in healthcare. The European Commission, for example, has established ethical guidelines for trustworthy AI [6]. These guidelines define standards for responsible AI development and promote international collaboration on beneficial AI solutions. Some key aspects of trustworthy AI include privacy and data governance [7] ensuring individual control over personal data without harm; diversity, promoting accessibility for all human capabilities; societal and environmental well-being, contributing to social change and sustainability; transparency, ensuring AI systems are traceable; and accountability, ensuring responsibility for AI outcomes. Similarly, Global Affairs Canada hosts an overview of two AI-related efforts in the Canadian public service: public consultation on AI and human rights and public engagement and collaboration in the compilation of an algorithmic impact assessment [8]. These efforts provide context for nationwide discussions on data and digital transformation and future initiatives aimed at more inclusive AI governance in Canada.

While several mechanisms exist to control and manage ethical-related issues, the most common way to ensure this principle is through checklists [9]. For example, Madaio et al. designed a checklist for fairness in AI [10]. The issue that is raised in the ethical AI checklists is the absence of comprehensive, technical, and implementable activities to integrate ethical principles into AI development. Furthermore, using frameworks was another solution; for instance, in Europe, legislative frameworks on crucial aspects like data protection have resulted in various regional, local, and national strategies for managing data [4]. This has led to the creation of the General Data Protection Regulation (GDPR), which promotes a more unified approach across the European Union (EU).

Despite these initiatives and guidelines, including those from the WHO on AI ethics and governance [11] and European strategy of digital health [12] significant ethical concerns persist. These include well-documented issues such as lack of transparency and accountability [13], algorithmic bias and [14, 15], and privacy issues in healthcare [16, 17]. These ethical concerns impact AI’s further advancement and adoption, particularly in the healthcare industry, where technology must comply with regulations, laws, and privacy standards to preserve the general welfare [18].

Beyond practical guidelines, the ethical deployment of AI in healthcare should be based on established theories. For example, Principlism includes autonomy, beneficence, non-maleficence, and justice, which provide a basic framework for evaluating AI systems [19]. Virtue ethics focuses on the character and virtues of the individuals involved [19], for example, it includes the development of ethical AI systems by fostering virtues such as trustworthiness, responsibility, and integrity. These theoretical lenses help to structure the analysis of ethical concerns or strategies while deploying or using AI in healthcare.

Recently, different studies [18, 20] have begun to examine both the ethical challenges and solutions for AI in healthcare. However, there are limited studies that comprehensively examine the current state of AI systems in the context of ethical concerns and strategies for implementing them in healthcare systems. Emerging technologies like AI demand continuous ethical implementation, particularly in critical domains like healthcare. Various studies have addressed this issue from different perspectives. For instance, Goirand et al. [21] conducted a scoping review of 33 articles to evaluate the implementation challenges and strategies of AI in healthcare, using an ethics framework. Their findings highlighted limited adoption of ethics frameworks and a significant gap in reporting on the practical implementation of such frameworks in AI-driven healthcare. Similarly, Morley et al. [22] reviewed 147 articles, mapping ethical issues across six levels of abstraction: individual, interpersonal, group, institutional, societal, and sectoral. While their work identified critical ethical challenges, it underscored the need for further exploration of solutions to address these concerns effectively.

Karimian et al. [4] analyzed 18 studies on the ethical principles of AI in healthcare, emphasizing fairness, autonomy, explicability, and privacy. Their work identified the necessity for designing and deploying AI ethical principles, although it provided limited practical solutions. Mennella et al. [23] examined a narrative review of ethical and regulatory challenges in digital healthcare, offering recommendations for stakeholders involved in AI decision-support systems. Similarly, Fan Li et al. [18] conducted a systematic review, addressing ethical concerns and strategies for designing AI in healthcare, stressing the importance of incorporating ethical considerations to mitigate the risk of AI misinterpretation. Liaw et al. [24] offered ethical recommendations regarding the use of electronic health record (EHR) data and AI, focusing on equity, fairness, and trust. Despite their valuable contributions, the study lacked a detailed exploration of challenges across different stages of the AI lifecycle.

Similarly, Gerke et al. [20] addressed ethical and legal challenges in AI-driven healthcare, particularly issues related to transparency, bias, and accountability, but their work remained at a high level, with limited actionable strategies. Safdar et al. [25] provided an overview of ethical concerns such as privacy and data ownership, but did not propose comprehensive solutions. Lastly, Morley et al. [26] explored practical methods for translating ethical principles into practice, aiming to bridge the gap between theory and implementation. However, their work did not adequately address the unique ethical challenges specific to healthcare AI applications.

In summary, while existing research has catalogued individual ethical concerns such as fairness, privacy, and autonomy, or the lack of examination of the ethical issues on AI ethics in healthcare, analyses often treat these issues in isolation, overlooking their interdependencies and broader contextual implementation challenges. However, these issues are often analyzed in isolation, with insufficient attention to their interdependencies or the broader contextual challenges they posess. While prior studies provide valuable insights, there is limited work that systematically synthesizes recent empirical evidence on technology used and methodology followed, stakeholder involved in the implementation and use of AI in healthcares, linking ethical concerns implementation strategies of AI in healthcare.

This review aims to fill this gap. It contributes by:

1. Identifying and analyzing a broader, interconnected range of ethical concerns (including transparency, trust, bias, fairness, privacy, data security, accountability, patient autonomy, and regulatory challenges) from empirical studies.
2. Highlighting practical, actionable strategies for their mitigation, with a specific emphasis on post-deployment monitoring and auditing.
3. Synthesizing empirical evidence on the technologies, methodologies, and stakeholders involved in real-world AI healthcare systems.

The research question (RQ) guiding this study is as follows:

- *RQ1*: What are the challenges or concerns in implementing ethics in AI in healthcare?
- *RQ2*: What strategies are used to address ethical issues of AI in healthcare?

This study aims to examine the existing ethical challenges in implementing AI in healthcare and the strategies available to address these issues. The remainder of the paper is organized as follows: Section Research methodology describes the methodology, sectionResults presents the results, and SectionDiscussions and conclusion discusses the review findings. Finally, Sect. 5 provides the concluding remarks.

## Research methodology

This review was conducted using a rigorous methodology to ensure comprehensiveness and minimize bias. We adhered to the PRISMA (Preferred Reporting Items for Systematic Reviews and Meta-Analyses) protocol [27] and followed the eight-step framework proposed by Okoli [28]. These guidelines were instrumental in structuring the entire review process, from defining the research purpose and drafting the protocol to identifying inclusion or exclusion criteria, executing the search, extracting data, appraising article quality, and ultimately synthesizing the findings.

The foundational purpose of this study was to systematically report the application of concerns and strategies related to the implementation of AI in the healthcare domain. To achieve this, we formulated specific research questions that guide our study. Following the initial scoping of the review, the researchers convened regularly to develop a detailed protocol. This protocol served as a foundational document, outlining all subsequent actions to mitigate potential bias during publication selection and data processing. A defined protocol is essential for ensuring the reproducibility and consistency of the review process [29]. Consequently, our protocol included the research questions, a detailed literature search strategy, the inclusion criteria, a plan for assessing study quality, and the methodology for data extraction [30].

The inclusion and exclusion criteria for study selection were meticulously developed based on the research questions and were informed by a prior review [17]. To be included, articles had to focus on studies that addressed topics such as ethics, concerns, challenges, strategies, principles, and guidelines related to the intersection of AI and healthcare. Conversely, the exclusion criteria were if the study was a case study, surveys, or various forms of reviews (including scoping reviews, literature reviews, systematic reviews, and meta-analyses). Furthermore, any study that exclusively discussed AI in healthcare without specifically addressing ethical concerns or proposing relevant solutions was also excluded, as presented in Table 1. We revised and verified that the included studies fully met the inclusion criteria, ensuring full consistency between the criteria and the final selection.

**Table 1:** Inclusion and exclusion criteria

No.	Inclusion criteria	Exclusion criteria
1	Studies addressing ethics in AI in health domain	Studies exclusively focus on AI other than health domain
2	Studies discussing ethical aspects in healthcare	Studies exclusively discussing healthcare only without AI
3	Articles published in the English language	Articles are written in languages other than English.
4	Empirical studies, including journal articles, conference papers, and book chapters.	Secondary studies, such as surveys, different types of reviews, and meta-analyses; editorial papers, abstract papers, case studies, reports, position papers.
5	The article was published between 2019 and 2025.	Articles published outside the specified year range.

### Search strategy

The initial set of primary studies was identified using carefully selected search keywords. A comprehensive literature search was conducted across multiple databases, including Scopus, ScienceDirect, PubMed, and Web of Science, to retrieve relevant studies. The ScienceDirect, Web of Science, and Scopus databases were chosen for their access to high-quality, peer-reviewed literature and broad coverage [31]. PubMed was selected for its content in the largest medical database [32]. A total of 243 articles were identified using the search terms: ‘ethic*’ OR ‘moral*’ AND ‘concern*’ OR ‘challenge*’ AND ‘strateg*’ OR ‘principle*’ OR ‘guideline*’ AND ‘artificial intelligence’ OR ‘AI’ AND ‘healthcare,’ covering the period from 2019 to 2024, ensure our research is current and relevant, reflecting the latest developments, findings, and methodologies in ethical concern and strategies of AI [33]. The search process was carried out between April 18 and April 21, 2024.

### Selection process

**Fig. 1:**
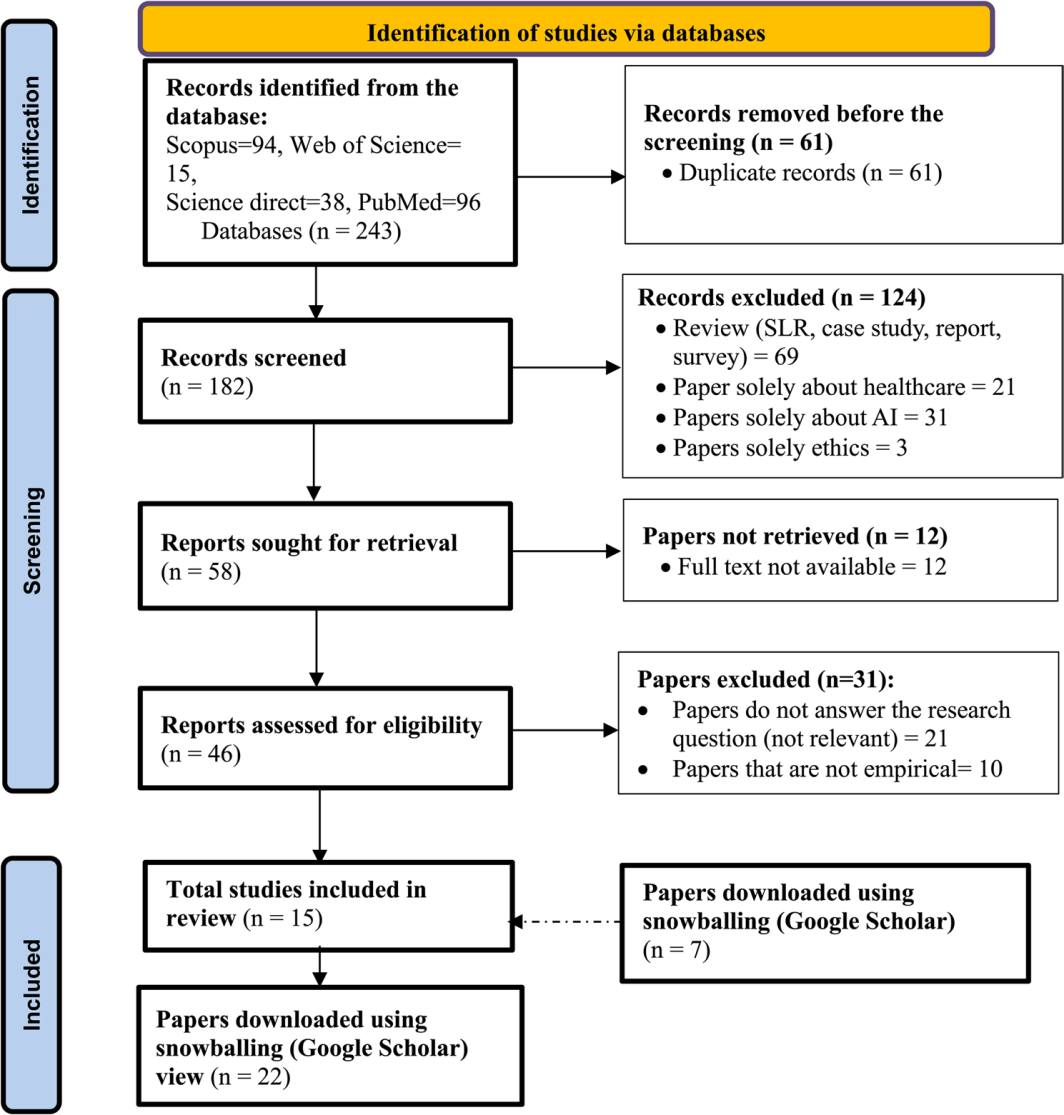
Flowchart of the study selection process

Following the identification of primary studies, the next step involved the selection of relevant articles. The studies were uploaded to the Rayyan AI tool to facilitate the screening process [34]. From a total of 243 papers, the first coder removed records (*N* = 61), leaving 182 studies for further screening. Both authors screened twenty studies (*N* = 11%) on title and abstract independently based on inclusion and exclusion criteria, and the inter-rater agreement was perfect agreement (Cohen’s Kappa = 0.89), as indicated by [35]. Conflicts were resolved through a consensus meeting, and the codebook was refined before the remaining articles were coded. Following that, the first author continued the screening, and 124 studies were excluded because they were systematic literature reviews, case studies, reports, surveys, or papers explicitly discussing AI, ethics, or healthcare. This left 58 studies for full-text retrieval. Of these, 12 full-text articles could not be accessible (requires subscription) and were therefore excluded. The remaining 46 articles were assessed for eligibility, and 21 studies were excluded for not addressing the research questions. Finally, 22 studies were included in the review. The selection process is summarized in Fig. 1. As shown, the majority of the articles were retrieved from PubMed and Scopus, followed by ScienceDirect, reflecting the relevant studies in these sources.

### Codification and data extraction

To make our study robust, we included additional studies (*N* = 7) through backward and forward snowballing [36]. Before full-text analysis, both authors collaborated to create a codebook [37]. The second coder initially developed a set of codes related to AI, ethical concerns, and strategies based on deductive coding from existing studies, which are presented in Table 2. This codebook was discussed and refined with the first author before data extraction commenced. Based on this deductive approach, the following categories were extracted from each study: descriptive statistics (e.g., year of publication), specific ethical concerns addressed, ethical strategies employed in AI applications, and the type of document analyzed. While the first author initially extracted the data, the second author verified the accuracy of the extracted information for 100% of the studies to ensure consistency and avoid risk of bias. The retrieved data were then thematically organized (e.g., themes of AI ethical strategies in healthcare) and synthesized within the framework of the review’s hypothesis [38].

**Table 2:** The codebook used in the study

CodeBook	Codes
AI	AI, Machine Learning, AI Models
Concerns	Issues, Challenges, Barriers,
Strategies	Principles, Frameworks, Guidelines, Protocols

## Results

This section presents the findings extracted from the primary studies, addressing the research questions, followed by a discussion of the results. The first subsection provides a general overview of the selected studies. The second subsection explores the ethical challenges faced by AI in healthcare, addressing Research Question 1. The final subsection examines the ethical strategies developed for AI in healthcare, addressing Research Question 2.

### Study characteristics

**Fig. 2:**
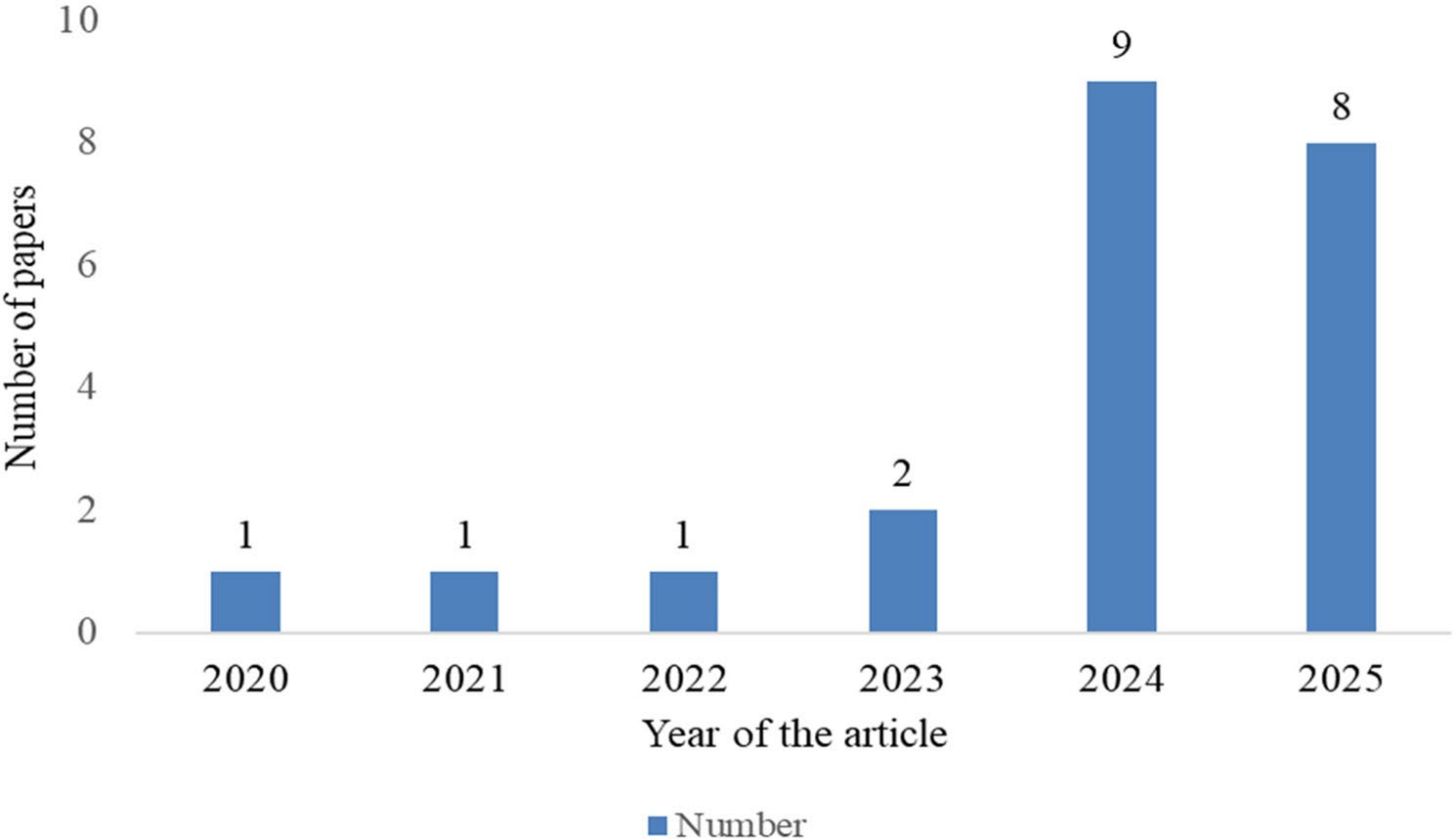
Selected articles based on the year proportion

After thorough analysis, while the dataset includes earlier work from 2020 (*N* = 1), 2021 (*N* = 1), 2022 (*N* = 1), and 2023 (*N* = 2), the majority of the studies were from 2024 (*N* = 9) and 2025 (*N* = 8). These account for over 77% of the total included studies, as shown in Fig. 2, and address AI-related ethical concerns and strategies. Regarding the publication venue, 20 studies were journal articles, while 2 were conference proceedings. The geographic distribution of the reviewed studies includes multiple studies from the United Kingdom (*N* = 3), Germany (*N* = 3), Turkey (*N* = 2), and the Netherlands (*N* = 2), alongside focused research in Canada, Sweden, Singapore, Denmark, Saudi Arabia, Australia, and the United States (specifically Florida) with a single study each. On a broader scale, three investigations were conducted within multinational frameworks: one including Europe, North, and South America; another involving the MENA region (specifically Jordan, Libya, Lebanon, Egypt, Palestine, Saudi Arabia, and Kuwait); and an international study primarily covering Switzerland, Germany, the UK, the USA, and the Netherlands. Additional data collection occurred at the European Congress of Radiology in Vienna, while one study location was not specified.

### Study setting and domain context

The context and domain of included studies were focused on different facets of high-stakes clinical implementations, such as radiology [39, 40] and medical imaging [41], cardiology, like ICD implantation for sudden cardiac death prevention [42], stroke medicine [43], and digital pathology for the early detection of Barrett’s Oesophagus [44], to the specialized practice areas. These areas includes nursing [45], pharmacy [46], emergency medical services [47], and mental health [48] with a specific focus on suicide prevention. Beyond specific clinical specialties, the context includes broad applications of Clinical Decision Support Systems [49], mortality prediction in emergency departments [50], and the use of diagnostic AI tools like digital scribes and virtual humans [51]. Furthermore, the studies explore the sociotechnical and ethical dimensions of AI, covering neuroscience data repositories [52], public health initiatives like COVID-19 monitoring [53], patient-centered care [54], and the integration of aging-in-place robotics and symptom checkers for older adults [55].

### Types of AI technology used and end users

Across the 22 studies, several Machine Learning (ML) and Artificial Intelligence (AI) technologies were used in different aspects of the health domain like in prediction, assisting with the technology. These include general data-driven methods [39, 56] and predictive analytics [50] to specialized deep learning architectures like neural networks and computer vision for medical imaging and pathology [44, 51, 57]. The integration of Natural Language Processing (NLP) and speech-to-text capabilities is also used in applications like virtual assistants, digital scribes, and portal messaging. Notably, the the the reviewed studies further reflected a further explored Explainable AI (XAI), utilizing frameworks such as SHAP, LIME, and ELI5 to ensure transparency, while others used emerging tools like Large Language Models (LLMs), rule-based expert systems, and Big Data analytics to support both diagnostic accuracy and operational clinical decision-making.

In-terms of end users involved in the implementation or usage of AI technology, in the reviewed we identified several stakeholders, including frontline healthcare professionals, such as physicians [53, 58, 59], clinical nurses [45], radiologists [41], pathologists [44], and pharmacists [46] and specific patients and the general public [54], such as older adults and individuals at risk of sudden cardiac death. The research also provided the perspectives of AI developers, researchers, and technologists who design the algorithms [48, 52], as well as hospital administrators, managers, and policymakers responsible for institutional implementation and governance [39, 48, 50, 51] Furthermore, the inclusion of ethicists, regulators, lawmakers [40], and family caregivers underscores a holistic approach to AI adoption.

### Methodological framework

In the reviewed study, different methodological approaches were used, for example, Qualitative approach, 62% (*N* = 13) of the total sample. Within this category, semi-structured interviews emerged as the primary instrument of choice (*N* = 9), often used to obtain stakeholder perspectives. Other qualitative designs included focus groups (*N* = 5) and participatory workshops (*N* = 3), which frequently integrated scenario-based or co-design principles to explore complex ethical or practical frameworks. Mixed-methods were the third method used 19% (*N* = 4) of the total, showcasing integration of data streams such as Delphi studies, the development of clinical checklists, and the pairing of quantitative ML experiments with qualitative case studies. In contrast, quantitative methods were less frequent, with only 10% (*N* = 2) of the studies, both of which used cross-sectional surveys. Finally, Theoretical and conceptual analysis made up the remaining 9% (*N* = 2), focusing on ethical modeling, and framework development.

### Ethical concerns or challenges in implementing AI in healthcare

**Fig. 3:**
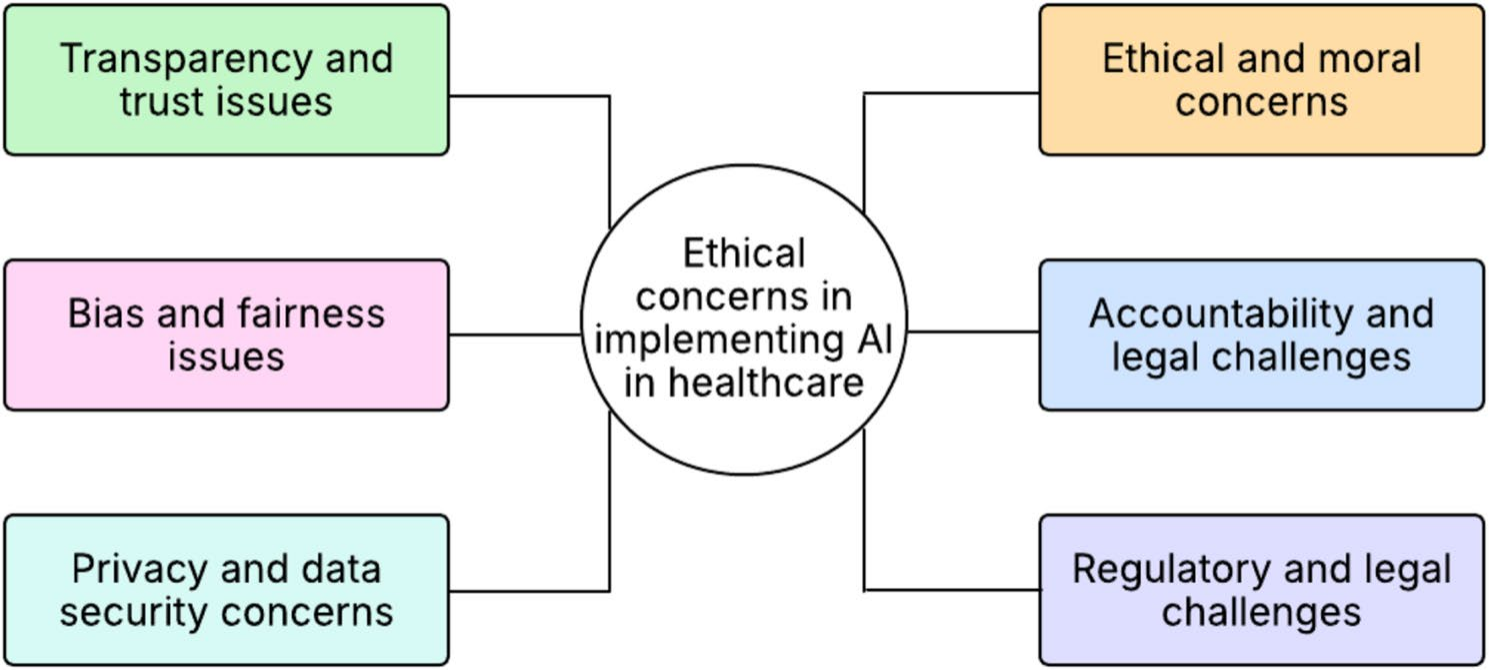
Ethical concerns in the implementation of AI in healthcare

Our analysis of empirical studies identified several challenges in health care in the implementation or usage of AI. We framed the extracted issues into seven key themes, as indicated in Fig. 3.

#### Transparency and trust issues

Several studies emphasize the critical importance of transparency and trust in the design, development, and deployment of AI systems for healthcare. Notably, six studies highlighted challenges associated with opaque AI systems [39], particularly when healthcare professionals struggle to interpret and explain AI-driven decisions. Recent studies show that when AI systems are not very transparent (not easy to understand), doctors have trouble explaining the AI’s results to patients and using these systems properly in their daily medical work [40, 41, 44, 46, 55, 59]. Explainability therefore continues to emerge as a key ethical concern in healthcare AI [47]. The absence of transparency in AI systems undermines trust and diminishes ownership of the data used [57, 60].

Recent evidence further indicates that insufficient transparency regarding data usage and decision-making processes intensifies trust concerns among both healthcare professionals and patients, especially in diagnostic and decision-support contexts [46, 51, 53, 60]. The studies also indicate that many existing algorithms lack transparency in AI systems of the health sector, such as data inclusion and exclusion criteria, and decision-making processes. Healthcare providers also report difficulties in managing the complexity of AI models and understanding how outputs are generated [42, 54], which raises concerns about accuracy, reliability, and the potential for AI systems to supplant human judgment [50]. For physicians, the black-box nature of AI systems may erode professional understanding and authority, ultimately undermining patient trust [42, 58].

#### Bias and fairness issues

AI systems in healthcare raise significant concerns regarding bias and fairness [39, 48]. Bias in AI systems disproportionately impacts disadvantaged groups, including women and racial minorities, leading to inaccurate diagnoses, unfair treatment recommendations, and a worsening of existing health disparities. For instance, biased datasets can result in AI algorithms underperforming for specific populations or failing to account for language and cultural variations [47, 52]. In addition [40, 60], show that biased training data and model design can result in AI underperforming for certain populations or failing to account for demographic, cultural, or clinical variations, which can worsen inequities in healthcare delivery.

Algorithmic bias may influence clinical predictions and decision-making, generating outcomes that do not fairly represent all patient groups [39, 48]. Even well-performing AI systems may produce biased results if the underlying datasets are incomplete, unrepresentative, or fail to reflect the diversity of the patient population [43, 55, 60]. Additionally, historical patient data often requires extensive preprocessing, such as reformatting and manual validation, before being incorporated into AI systems. This process may result in misrepresentation or data loss, further introducing bias [39].

#### Privacy and data security concerns

AI systems often process vast amounts of sensitive patient data, raising significant privacy and data security concerns. The collection, sharing, and storage of patient data for AI training can expose information to risks such as unauthorized access, breaches, and unintended disclosure, even when datasets are anonymized [40, 45, 51, 59]. Finding individuals from apparently anonymized datasets remains a practical concern, highlighting the need for careful data security and protection measures [46, 51, 54, 58].

Ethical challenges in the clinical application of AI include safeguarding patient privacy, ensuring informed consent, and addressing equitable access [53]. Protecting patient information from misuse and unauthorized access is a critical concern that requires robust security measures and oversight [41, 58].

#### Accountability and responsibility issues

The integration of AI into healthcare introduces significant challenges in accountability and responsibility. A core concern is ensuring that AI serves as a supplement to human judgment and decision-making in medical care, rather than a replacement [40, 45]. However, the advanced capabilities of AI systems, particularly when their accuracy surpasses that of human evaluators, create ambiguity in assigning responsibility for both positive and negative outcomes [41, 44]. This lack of clarity extends to legal liability. The autonomous nature of AI and the difficulty in identifying the responsible party when harm occurs make it challenging to determine legal culpability. Moreover, the complexity of how these systems arrive at decisions complicates efforts to establish clear accountability and navigate approval processes [49].

The complexity and ‘black-box’ nature of many AI models further complicate the assignment of legal and moral responsibility, making it difficult to identify the accountable party in cases of harm [44, 55]. AI may fragment accountability, as multiple stakeholders, including developers, clinicians, and administrators, are involved in decisions. Improving model transparency through XAI and considering sociocultural factors, like training and daily workflow integration, are also crucial in health care AI applications [43, 57].

#### Ethical and moral concerns

The integration of AI into healthcare brings forth numerous ethical and moral challenges. Attempting to formalize ethics and morality into rigid frameworks for AI development often results in inconsistencies, ambiguities, and incomplete models, which may hinder the responsible implementation of these technologies [40, 49]. Moreover, cultural and social values complicate the creation of universal ethical standards, as norms and expectations vary significantly across different societies and geographic regions.

Beyond these foundational issues, the ethical trade-offs involved in deploying AI in healthcare require being carefully navigated. Striking a balance between patient-centered care and profit-driven motives, preserving patient autonomy while preventing over-dependence on AI systems, and safeguarding data privacy without compromising operational efficiency are just a few of the complex considerations [43]. Some patients may feel uneasy about trusting AI or may prefer not to involve it in their treatment plans, as it might conflict with their values and personal preferences [42].

Current ethical guidelines often focus narrowly on the individual-level impact of AI, overlooking broader social and institutional ramifications [56]. However, the potential for AI to reshape social structures and potentially lead to health inequities demands critical attention. Addressing these broader implications is essential to ensuring that AI adoption in healthcare promotes equity and inclusivity.

The integration of AI also poses a risk of diminishing the importance of shared decision-making and informed consent, both of which are critical to respecting patient autonomy [42]. Shared decision-making relies on open dialogue and mutual understanding between patients and healthcare providers, a process that could be disrupted if AI systems overly mediate or dictate clinical decisions. Additionally, he opaque nature of AI systems can make it difficult for patients to understand how their data is used, potentially undermining informed consent and the collaborative patient-clinician relationship [40].

#### Regulatory and legal challenges

Developing appropriate policies and legislation to govern the use of AI in healthcare is critical to ensuring its safe and ethical integration [50]. However, the autonomous and complex nature of AI systems creates challenges for regulation, and many AI products lack sufficient public transparency regarding development, ethical considerations, and deployment risks [40]. These gaps highlight the need for clear legal requirements to ensure accountability, safety, and ethical compliance.

### Ethical strategies and considerations in using AI in healthcare

**Fig. 4:**
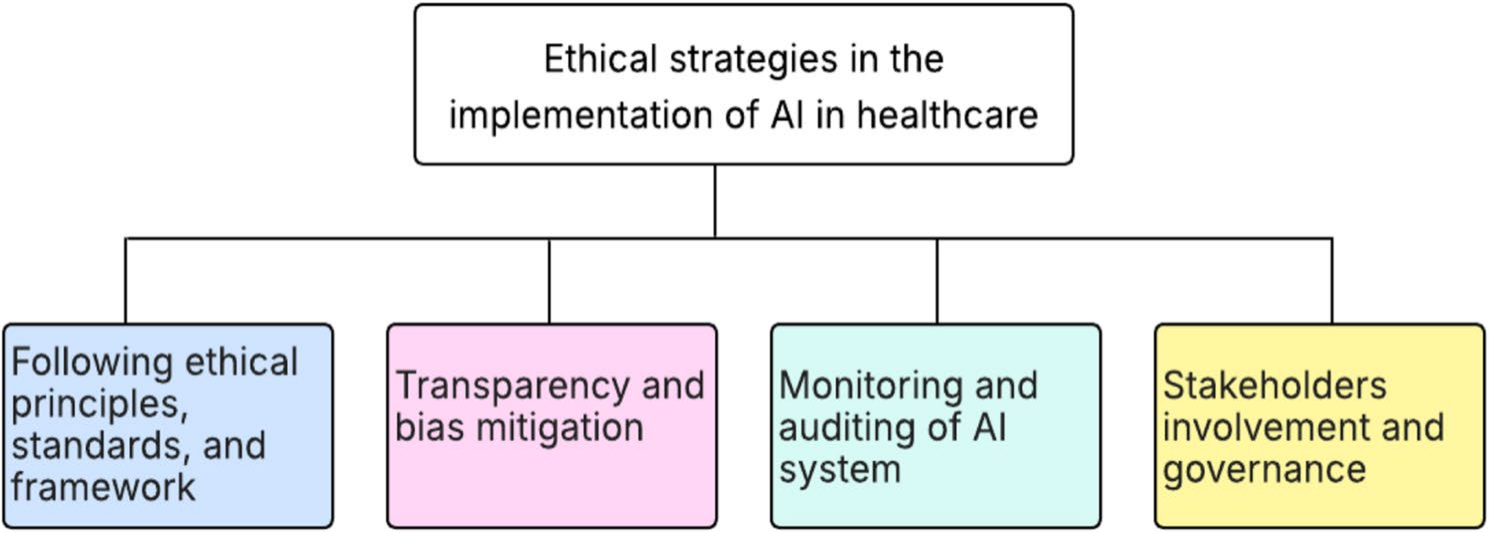
Ethical strategies of AI in healthcare

Based on our analysis of the selected studies, we identified four key ethical strategies and considerations for implementing or using AI systems in healthcare. The summarized categories are presented in Fig. 4. The operational details of these ethical strategies are depicted in Table 3.

**Table 3:** Operational details of ethical strategies

Strategy	Operational actions supported by the included studies
Ethical frameworks	Ethics checklists [48], impact assessments [47]
Transparency and Bias Mitigation	XAI tools in emergency calls [47], documentation of datasets [52], bias mitigation [52]
Monitoring & auditing	post-deployment performance audits [47], quality assessment [40].
Stakeholder involvement	participatory design & implementer interviews [39]

#### Following ethical principles, standards, and framework

Adhering to ethical principles, standards, and frameworks is the first strategy raised to ensure the responsible and effective use of AI in healthcare. Two studies indicate that developing AI systems with explicit attention to ethical principles, human values, and patient safety helps mitigate potential biases and ethical risks throughout the AI lifecycle [40, 45].

Several studies describe practical ways to apply ethical principles during AI development and deployment. For example, in study [48], presents an ethics checklist designed for suicide prevention and mental health, and notes that many of its items are also applicable to other healthcare AI contexts. In clinical settings, study [47] applied the Z-Inspection impact-assessment process to evaluate AI systems in practice, helping identify concrete ethical risks and trade-offs related to safety, transparency, and accountability before and during deployment.

AI systems should be developed with a focus on safety, efficacy, explicability, human values, and ethical principles [55, 59]. While consulting AI experts in ethics during the development process is essential to address potential biases and limitations [48], this proactive approach helps identify and mitigate ethical risks early in the AI lifecycle.

Adhering to ethical and governance frameworks during AI development and deployment helps ensure operations remain safe, fair, and patient-centered [40]. Robust oversight, clear regulations, and transparent data practices promote accountability, maintain data integrity, and foster trust in AI systems, enabling responsible and equitable healthcare applications [59].

The ethical evaluation of AI applications, such as those used for mortality risk prediction, supports the principles of beneficence and non-maleficence. While such systems assist clinicians in producing favorable outcomes for patients by balancing potential benefits against risks [50] Explainable AI (XAI) techniques are particularly important for enhancing interpretability. By clarifying how AI models arrive at their decisions, XAI opens the black box of AI decision-making [47].

#### Transparency and bias mitigation

This involves mitigating bias and ensuring AI transparency and interpretability to enable the safe and effective use of AI in healthcare. Transparency in AI promotes trust and facilitates the adoption of AI tools by healthcare providers and patients, making it a cornerstone of ethical and effective AI deployment in clinical settings.

Several studies describe practical steps to improve transparency and reduce bias in healthcare AI. In emergency call systems, dispatchers must understand why the system raises an alert, and suggest using simple explanation tools (e.g., highlighting relevant words, voice tone, or breathing patterns) so users can detect errors and potential bias [47]. In addition, studies stress the importance of documenting how datasets are constructed and which populations are represented [52]. Bias can also be reduced by training models on diverse datasets and accounting for ethnic and regional differences in health records [52].

Healthcare professionals are responsible for promoting patient autonomy, minimizing harm, and ensuring that AI systems are transparent, ethical, and useful in clinical practice [43, 51, 60]. Explicability, which refers to the necessity for a clear understanding of AI data and algorithm functioning, is another critical aspect for ensuring reliable AI systems [50]. Transparency not only builds trust but also enhances the usability and adoption of AI systems by clinicians and patients [45, 54].

Several studies, such as [47] highlighted the need for clear guidelines and systematic training for healthcare professionals on how to interact with AI systems and interpret their predictions. Developing systematic strategies and ethical frameworks for the safe use of AI in clinical practice is essential. Proper training for healthcare professionals and the establishment of forums to discuss AI-related ethical issues were also identified as critical components [50]. Models of clinician–algorithm collaboration, such as peer review, triage, and auditing approaches, are recommended to ensure safe and effective AI use in clinical practice [53]. These models benefit from transparent and explainable AI systems, which support a collaborative approach between clinicians and AI [43].

Transparency in AI can be achieved through improved communication between developers and end-users, access to source code, documentation of data usage, and outlining potential outcomes in easily understandable terms [56]. It involves making AI processes visible and understandable, ensuring coherence, and providing clear explanations through text or visuals. This enhances accountability, trust, and the ability to validate decisions made by AI systems.

Additionally, embedding ethical principles and bias mitigation strategies into the initial design of AI systems promotes health and well-being [52]. Ensuring high-quality, diverse, and representative datasets is particularly important for reducing algorithmic bias and improving model generalizability [39, 47]. Training on unbiased data enhances traceability and minimizes algorithmic biases [50]. Addressing bias requires careful curation of datasets to minimize risks related to ethnicity and other socio-demographic factors. Adhering to governance standards, such as appropriately including or excluding migrant populations in local repositories, promotes fairness and equity in data-driven decision-making [52]. These measures uphold ethical standards in data management and analysis.

#### Monitoring and auditing of an AI system

The third strategy involves continuous monitoring and auditing of AI systems. This emphasized the importance of monitoring and auditing AI systems in healthcare to maintain ethical standards and fairness. Marco-Ruiz et al. [39] emphasized the importance of implementing mechanisms to continuously assess AI performance and detect any potential degradation over time. Regular audits also help identify biases and ensure algorithms remain fair and effective across diverse populations [40].

Post-deployment evaluation is also emphasized in several studies. Study [47] assessed safety by combining retrospective data analysis with real-time clinical trials, allowing problems to be detected as the system was used in practice. Embedding AI evaluation into routine quality-assurance processes, including outcome monitoring, bias assessment, and performance checks, helps ensure that AI systems remain safe, effective, and can be adjusted or withdrawn if risks emerge [40].

Effective monitoring and auditing of AI systems is crucial, as older adults’ trust depends not only on technical performance but also on transparency, human oversight, and the social context in which these systems are deployed [55]. A comprehensive and continuous evaluation framework not only mitigates risks and biases but also ensures the long-term ethical integrity of AI systems. By addressing these aspects proactively, organizations can maintain trust and accountability while adapting to the dynamic requirements of healthcare applications [47].

#### Stakeholders’ involvement and governance

Stakeholder involvement, encompassing AI developers, end-users, practitioners, and clinicians, emerged as a significant theme in the ethical governance of AI systems. This emphasized the critical role of stakeholder engagement in ensuring ethical AI practices. Involving stakeholders fosters accountability in AI development and deployment processes [41, 44, 46]. Incorporating diverse perspectives and addressing stakeholder concerns are key to safeguarding the integrity of AI systems. This involves proactive discussions with stakeholders and organizations to address potential ethical dilemmas, build trust, and promote responsible AI practices that align with societal expectations.

Engaging stakeholders during the design phase of AI projects is particularly important [39, 47]. Participatory approaches, such as including minority groups in the development process, ensure that their needs and perspectives are considered [54]. This inclusive strategy enhances the ethical governance of AI and increases public acceptance and support for AI technologies across various domains [49, 51].

At the operational level, studies stress that ethical AI depends on involving the right people throughout development and deployment. In [39], clinicians, vendors, and policymakers are engaged during procurement and implementation to ensure that systems fit clinical practice and policy requirements. Including end users in testing and feedback also helps identify risks that may not be visible to developers alone. In addition, public health and fairness-focused studies emphasize the importance of involving patient representatives and minority groups so that AI systems do not overlook vulnerable populations and can better reflect real-world diversity.

## Discussions and conclusion

The results highlight ethical concerns in implementing AI in healthcare, emphasizing transparency, bias, data privacy, accountability, and patient autonomy. A significant finding was the emphasis on the black box nature of AI systems, which undermines transparency and trust among both healthcare professionals and patients. This aligns with previous studies [42, 61, 62] that have called for Explainable AI as a solution to enhance interpretability and foster trust. Moreover, the persistence of algorithmic bias, as evidenced in the studies [48, 63], underscores the urgent need for diverse and representative datasets. The surge in recent literature (2024–2025) within our analysis signals that the field has moved from theoretical debate to confronting the practical governance challenges of real-world AI integration across diverse clinical settings.

While ethical principles and frameworks are provided as solutions to the challenges of AI in healthcare, our analysis reveals that these strategies can often exist in tension with one another, requiring careful trade-offs. A central conflict lies between the goals of promoting patient autonomy [64] and the drive toward increasing automation [65]. We observed that when AI becomes autonomous, and its accuracy surpasses human evaluators, there is a risk that clinical decisions guided by algorithms may override a patient’s personal values and preferences [66]. The push for automated efficiency might reduce the time available for shared decision-making, which is fundamental to respecting patient autonomy. Thus, a strategy focused on improving clinical workflow through automation may weaken the physician-patient relationship and the patient’s right to make informed choices.

Another trade-off exists between the strategies of enhancing transparency and safeguarding patient privacy. While XAI and open access to data are essential for building trust and mitigating bias, this need for transparency can conflict with the imperative to protect sensitive patient information. As identified by [61], the extensive data sharing required for auditing and improving AI models increases the risk of re-identification. Similarly, mitigating bias with vast, diverse datasets [50] expands the attack surface of potential breaches [34]. The methodological predominance of qualitative approaches (e.g., interviews, focus groups) in the reviewed studies effectively captures the stakeholder tensions but also highlights an evidence gap that requires studies that could measure the prevalence of these ethical risks and the efficacy of proposed interventions.

This study has several theoretical implications. The tension between transparency and privacy, for example, reflects the conflict between deontological ethical theory (adherence to rules and duties) and consequential risk (of data breach), which is the ethical theory of breach [19]. The focus of bias mitigation aligns with a justice-based framework, which demands equitable health outcomes.

Overall, the study offers a comprehensive analysis of the ethical landscape of AI in healthcare, categorising concerns into six themes and strategies into four thematic areas. This structural framework is relevant for stakeholders. However, the analysis also reveals a strategic imbalance; for example, while transparency and bias mitigation are heavily emphasized, foundation strategies such as continuous monitoring and stakeholder involvement receive less attention. This suggests an over-reliance on technical solutions at the expense of robust governance processes. Future studies, therefore, develop and evaluate procedural frameworks such as a participatory design protocol and audit mechanisms, which are necessary to translate ethical principles into practices that ensure AI adoption safeguards patent welfare and trust.

Future studies should engage with ethical theories to develop a robust and theoretically grounded framework for AI governance in healthcare, while interdisciplinary collaboration between ethicists, clinicians, and AI developers is needed to map the relevant theoretical principles into context-sensitive guidelines for the AI implementation.

## Supplementary Information

Supplementary Material 1.

## Data Availability

All data generated or analysed during this study are included in this published article and its supplementary information files.
